# Supplementary material for: Transmission of cervid prions to humanized mice demonstrates the zoonotic potential of CWD
Source: Acta Neuropathol. 2022 Aug 22;144(4):767–84. doi: 10.1007/s00401-022-02482-9 (PMC9468132; doi:10.1007/s00401-022-02482-9)
Supplement: Supplementary file 1 — Supplementary file1 (PDF 1670 KB) [file 401_2022_2482_MOESM1_ESM.pdf]

Supplementary information

Transmission of Cervid Prions to Humanized Mice Demonstrates the Zoonotic Potential of CWD

Authors: Samia Hannaoui[1], Irina Zemlyankina[1], Sheng Chun Chang[1], Maria Immaculata Arifin[1], Vincent Béringue[2], Debbie McKenzie[3], Hermann M. Schatzl[1], Sabine Gilch[1*]

Affiliations:

[1] Department of Comparative Biology and Experimental Medicine, Faculty of Veterinary Medicine; Hotchkiss Brain Institute; University of Calgary, Calgary, Canada

[2] Université Paris-Saclay, INRAE, UVSQ, VIM, 78 350 Jouy-en-Josas, France

[3] Department of Biological Sciences, Center for Prions and Protein Folding Diseases, University of Alberta, Edmonton, Canada

*Corresponding author. Email: sgilch@ucalgary.ca

Phone #: 403-210-7578

Journal: *Acta Neuropathologica*

Supplementary Table 1. Transmission of CWD prions to tg650 transgenic mice overexpressing human PrP (129MM)

**Animal original ID#**	**CWD inocula**	**Days post infection (DPI)**	**Status of animal at euthanasia**	**Clinical signs of prion disease**	**Reason for euthanasia**	**IHC**	**RT-QuIC**			
							Brain	Faeces FH dilution: % of positive replicates		
								10^{-1}	10^{-2}	10^{-3}
#2-HcW-1-321	Wisc-1	882 dpi	Terminal clinical signs	Myoclonus, Weight loss, Hyperexcitability, Kyphosis, Hind-limb clasping, Rigid tail, Rough coat	Terminal	NA	Pos	12.5%	12.5%	12.5%
#2-HcW-1-322		791 dpi	-	No signs of prion disease	Humane endpoint	NA	Pos	Neg	Neg	Neg

#2-HcW-1-323		919 dpi	Terminal clinical signs	Myoclonus, Repeated weight loss and gain, Rigid tail, Kyphosis, Hind-limb clasping	Experimental endpoint	Neg	Pos	25%	Neg	Neg
#2-HcW-1-324		919 dpi	Subtle clinical signs	Myoclonus, Repeated weight loss and gain	Experimental endpoint	Neg	Pos	Neg	Neg	Neg
#2-HcW-1-325		789 dpi	Terminal clinical signs	Myoclonus, Repeated weight loss and gain, Rigid tail, Kyphosis, Hind-limb clasping, Mild ataxia, Paralysis	Terminal	NA	Pos	Neg	Neg	Neg
#2-HcW-1-326	Wisc-1	934 dpi	-	Myoclonus	Experimental endpoint	Neg	Inconclusive	Neg	25%	12.5%
#2-HcW-1-327		623 dpi	Terminal clinical signs	Myoclonus, Weight loss, Rigid tail, Kyphosis, Hind-limb clasping, Ataxia, Paralysis, Heavy breathing, Irresponsiveness, Gait abnormalities	Terminal	Neg	Inconclusive/Neg	75%	100%	75%
#2-HcW-1-328		934 dpi	Subtle clinical signs	Myoclonus, Heavy breathing	Experimental endpoint	**Pos**	Pos	Neg	Neg	12.5%

#2-HcW-1-329	Wisc-1	934 dpi	Subtle clinical signs	Myoclonus, Heavy breathing	Experimental endpoint	Neg	Pos	Neg	Neg	Neg
#2-HcW-1-330		213 dpi	-	No signs of prion disease	Found dead	NA	NA	NA	NA	NA
#2-HcW-1-331	116AG	882 dpi	Terminal clinical signs	Myoclonus, Weight loss, Hind-limb clasping, Gait abnormalities, Hind-limb paralysis	Terminal	NA	Inconclusive/Neg	12.5%	12.5%	12.5%
#2-HcW-1-332		919 dpi	Subtle clinical signs	Myoclonus, Gait abnormalities	Experimental endpoint	NA	Neg	Neg	Neg	Neg
#2-HcW-1-333		919 dpi	Subtle clinical signs	Myoclonus, Repeated weight loss and gain	Experimental endpoint	NA	Neg	Neg	Neg	Neg
#2-HcW-1-334		722 dpi	-	No signs of prion disease	Humane endpoint	NA	NA	Neg	Neg	Neg

#2-HcW-1-335	116AG	751 dpi	-	No signs of prion disease	Found dead	NA	Neg	Neg	25%	Neg
#2-HcW-1-336		934 dpi	-	Myoclonus	Experimental endpoint	NA	Neg	12.5%	Neg	12.5%
#2-HcW-1-337		736 dpi	-	No signs of prion disease	Found dead	NA	Neg	NA	NA	NA
#2-HcW-1-338		919 dpi	Subtle clinical signs	Weight loss, Kyphosis, Gait abnormalities, Myoclonus and hyperexcitable	Experimental endpoint	NA	Neg	12.5%	12.5%	Neg
#2-HcW-1-339		934 dpi	Terminal clinical signs	Myoclonus, Kyphosis, Gait abnormalities	Experimental endpoint	NA	Neg	Neg	Neg	Neg
#2-HcW-1-340		934 dpi	-	Myoclonus	Experimental endpoint	NA	Neg	Neg	Neg	Neg

Supplementary Table 2. Bioassay of white-tailed deer CWD isolates in tg650 mice

Inoculum	Prion clinical mice		No prion signs
	Terminal clinical signs	Subtle clinical signs	
Wisc-1	44.4%	33.3%	22.3%
116AG	28.6%	42.9%	28.5%
Neg CTR	0%	0%	100%

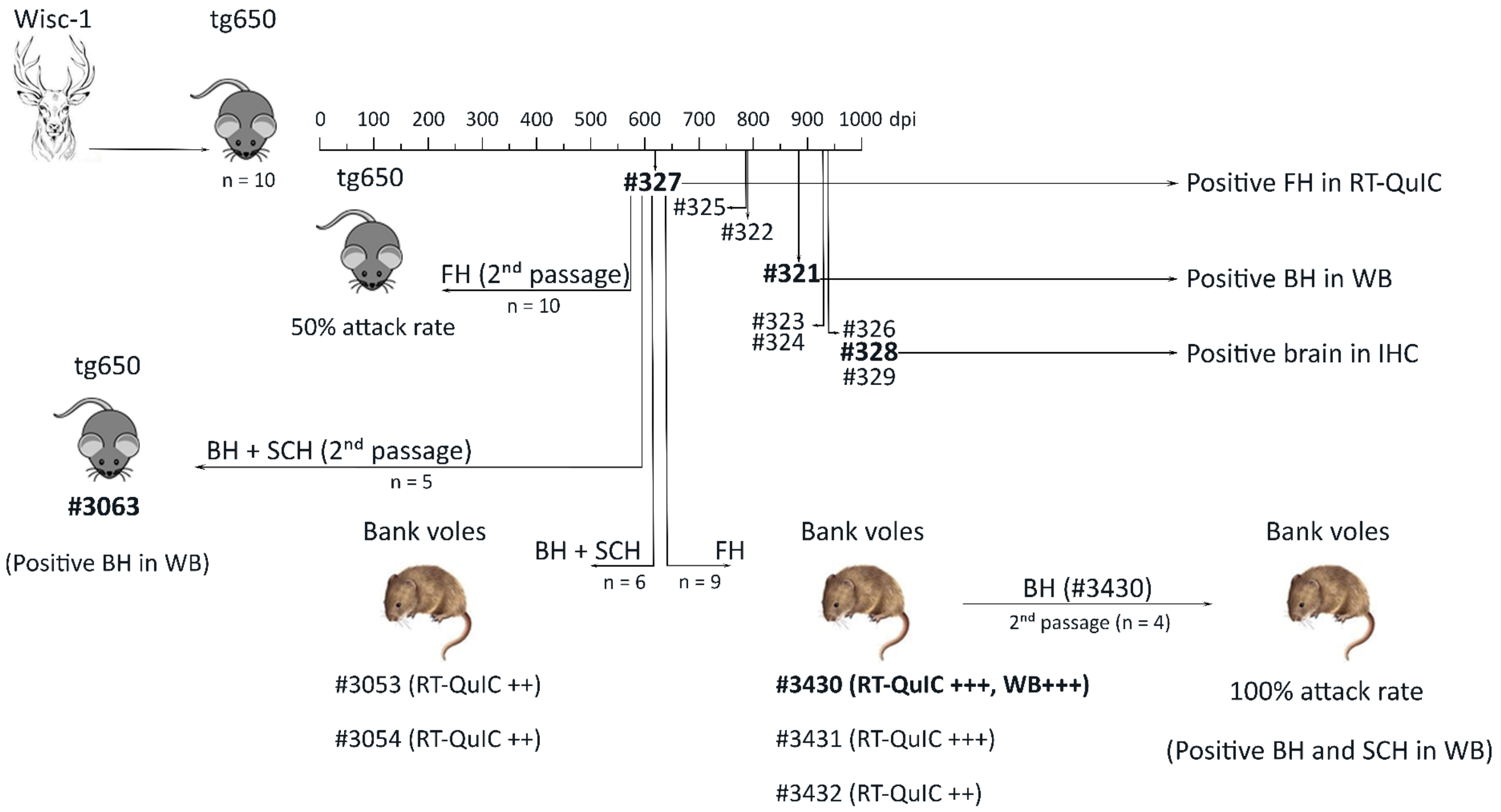

Supplementary Fig. 1

Scheme of the transmission study of WTD Wisc-1-CWD in tg650 and bank vole models

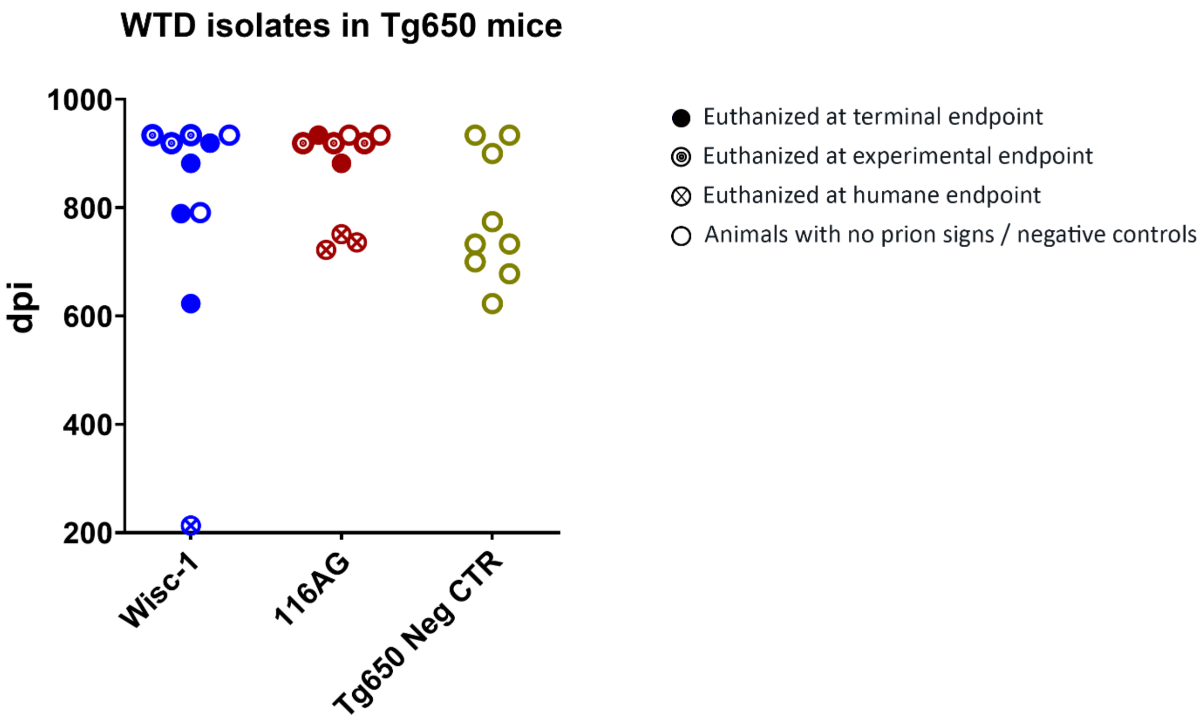

Supplementary Fig. 2

Transmission of WTD isolates in tg650 mice. Tg650 mice were inoculated with Wisc-1 (blue), 116AG (red), or not inoculated (age-matched control; green). Distribution of incubation period in tg650 mice is classified according to the status of mice at the time of euthanasia. Mice are defined with terminal clinical signs (full circles), subtle clinical (bullet circles), or no signs (open circles). Circles with crosses represent mice euthanized due to intercurrent diseases.

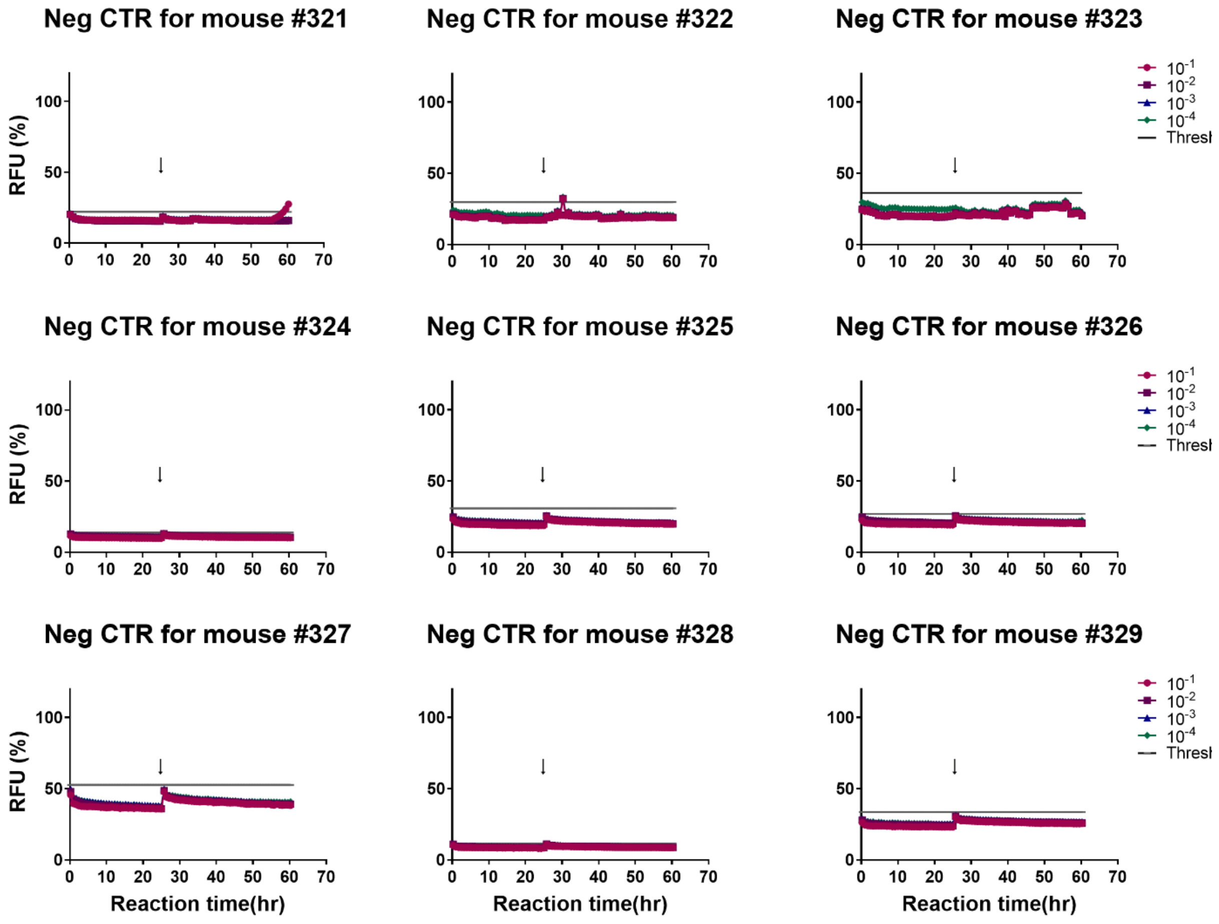

Supplementary Fig. 3

RT-QuIC analysis of BH seeding activity of age-matched tg650 controls used as internal negative controls for each Wisc-1-tg650 inoculated mouse. The graphs depict a representative RT-QuIC assay of brain homogenates from tg650 age-matched negative control mice. Twenty percent of brain homogenates were serially diluted (10^{-1} – 10^{-8}) and seeded in mouse rPrP substrate. The introduction of substrate replacement is indicated with a black arrow. The cut-off (threshold) was based on the average fluorescence values of negative control +5x SD.

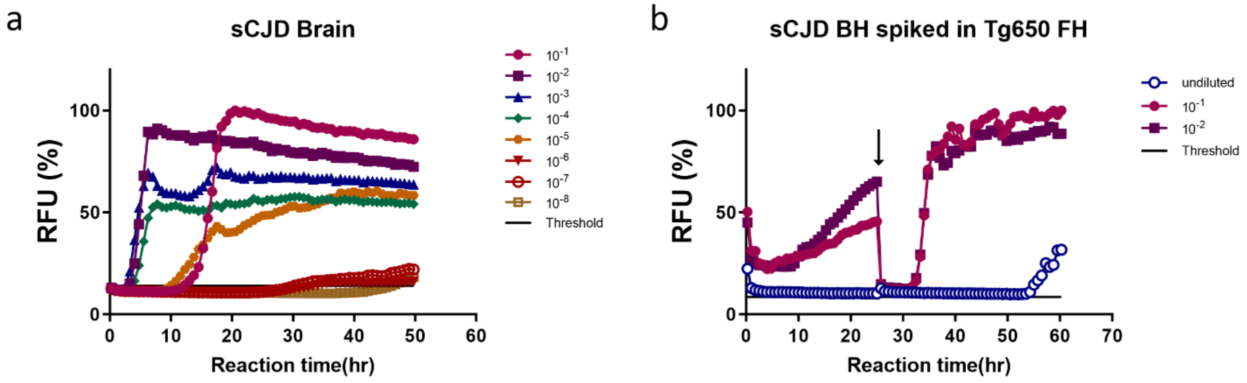

Supplementary Fig. 4

a. RT-QuIC analysis of sCJD BH prion seeding activity. The graphs depict a representative RT-QuIC assay of brain homogenates from an sCJD patient-MM1. Twenty percent sCJD brain homogenates were serially diluted ($10^{-1} – 10^{-8}$) and seeded in mouse rPrP substrate. **b.** RT-QuIC analysis of sCJD BH spiked in fecal homogenate of tg650 naïve mouse and seeded in mouse rPrP substrate. The introduction of substrate replacement is indicated with a black arrow. The cut-off (threshold) was based on the average fluorescence values of negative control +5x SD used in every assay.

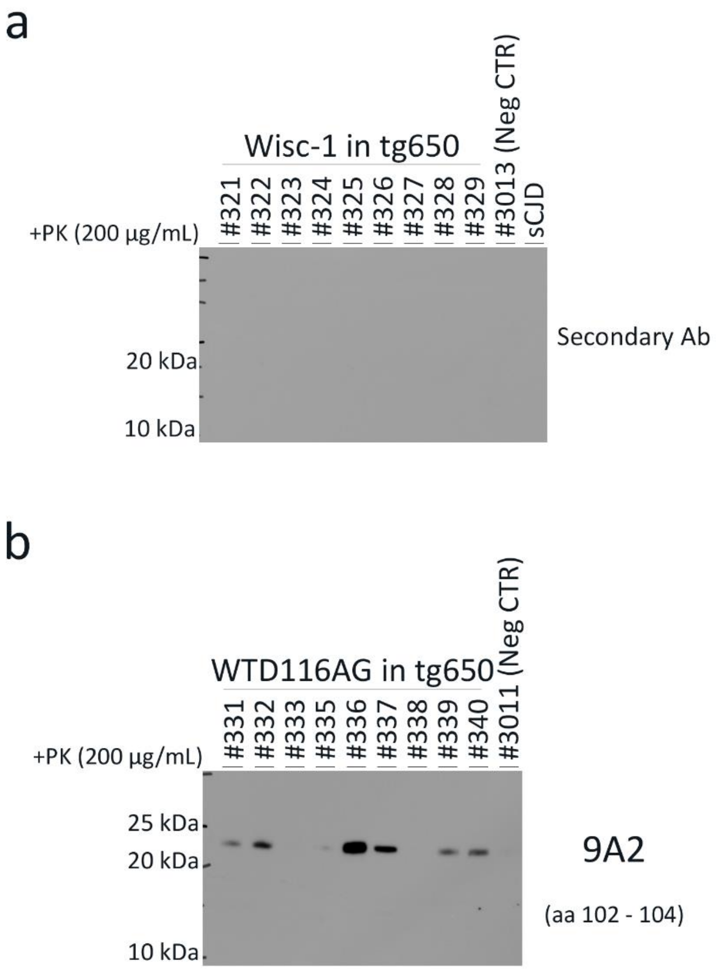

Supplementary Fig. 5

a. Western blot analysis of brain homogenates of tg650 mice inoculated with Wisc-1 isolate and digested with 200 µg/mL of PK using only horseradish peroxidase-conjugated goat anti-mouse IgG. **b.** Western blot analysis of brain homogenates of tg650 mice inoculated with 116AG isolate and digested with 200 µg/mL of PK using anti-PrP mAb, 9A2 (aa 102 – 104, bottom). A negative control, tg650 #3011 was also included in the western blot.

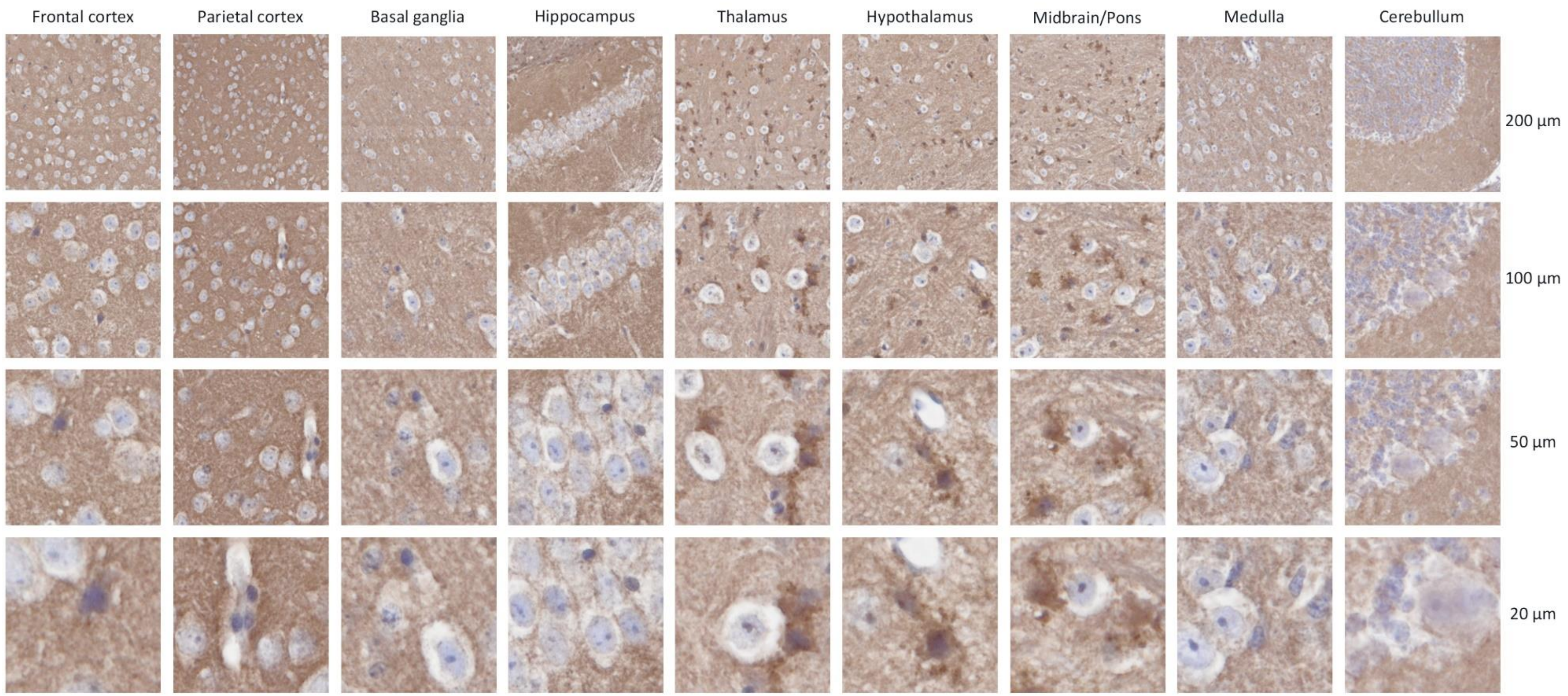

Supplementary Fig. 6

PrP^{Sc} staining of Wisc-1-tg650 inoculated mouse #328. Immunohistochemistry using PK digestion step shown in nine brain regions (frontal cortex, parietal cortex, basal ganglia, hippocampus, thalamus, hypothalamus, midbrain/pons, medulla, and cerebellum) demonstrates the specific immunostaining observed in the thalamus, hypothalamus and midbrain/pons areas as opposed to the nonspecific background staining seen in all other areas. Scale bars, 200 µm, 100 µm, 50 µm, and 20 µm

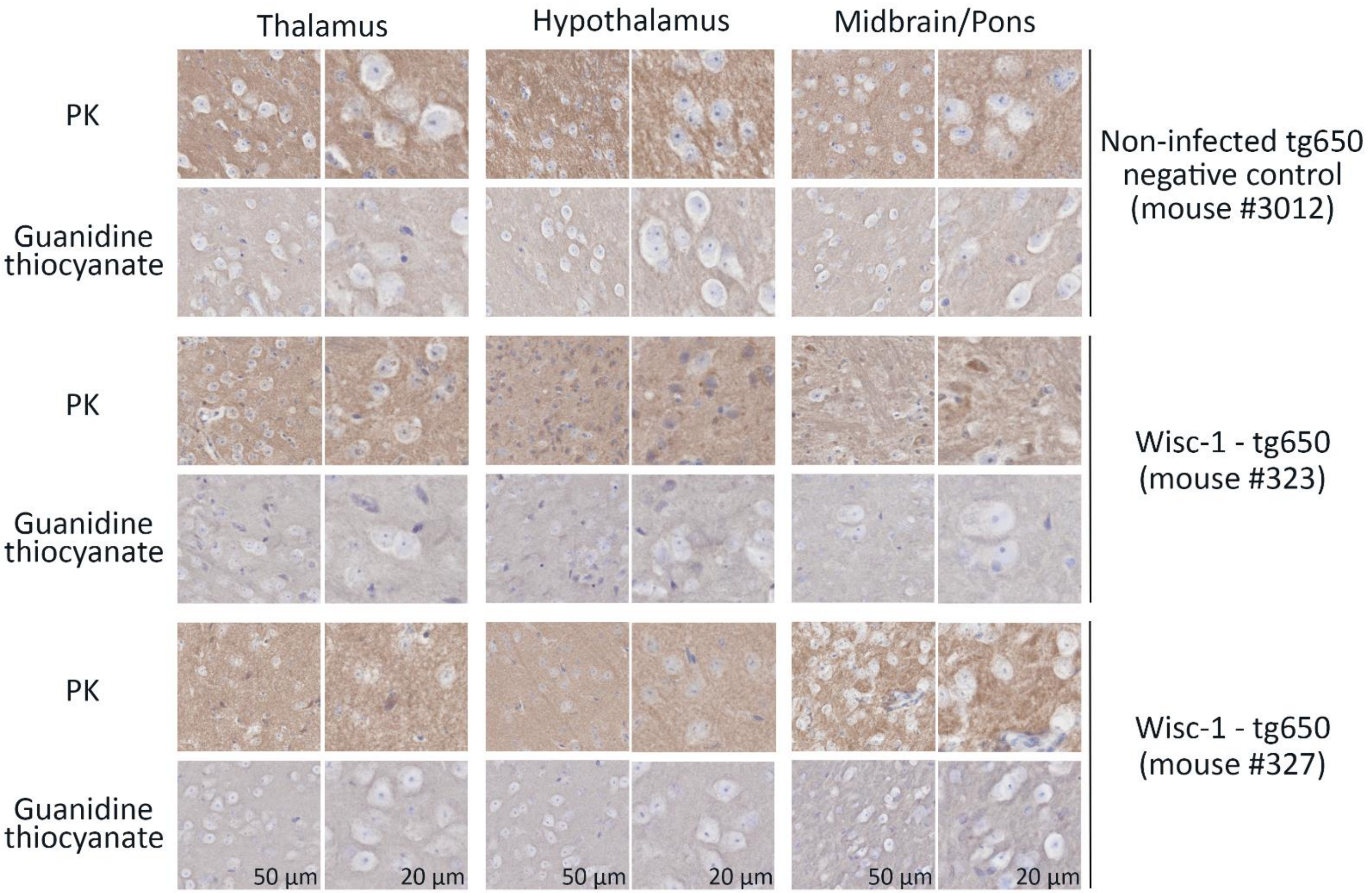

Supplementary Fig. 7

PrP^{Sc} staining of age-matched negative tg650 control, and Wisc-1 inoculated tg650 mice. Immunohistochemistry using either PK digestion (upper panels) or guanidine denaturation (lower panels) demonstrating the lack of PrP^{Sc} staining in the thalamus (left panels), hypothalamus (middle panels), and midbrain/pons (right panels) areas of tg650 negative control #3012 (upper panels), Wisc-1 inoculated tg650 mouse #323 (middle panels), and mouse #327 (lower panels). Scale bars, 50 µm, and 20 µm

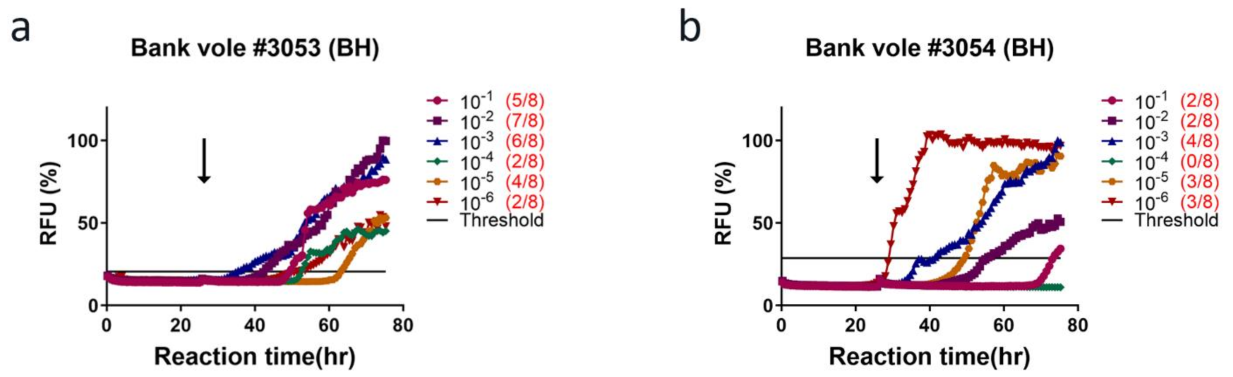

Supplementary Fig. 8

Transmission of Wisc-1-tg650 to bank voles. One percent brain/spinal cord homogenates from Wisc-1-inoculated tg650 mouse #327 was transmitted intracerebrally to bank voles. The curves depict a representative RT-QuIC assay of brain homogenates from bank vole #3053 (a), and #3054 (b). The cut-off (threshold) was based on the average fluorescence values of negative control +5x SD used in every assay.

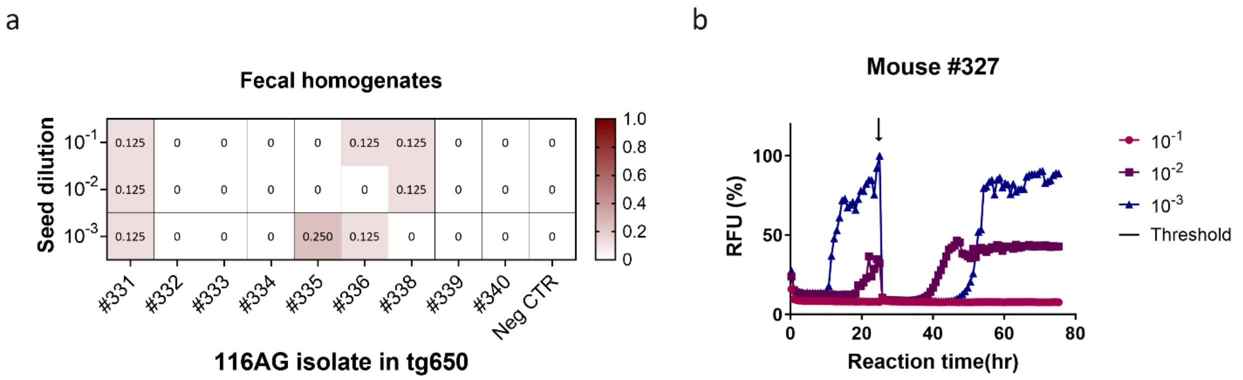

Supplementary Fig. 9

Prion seeding activity detected in feces of 116AG-inoculated tg650 mice. **a.** Summary of RT-QuIC analysis of prion seeding in the fecal material of 116AG inoculated humanized mice. The heat map indicates the percentage of positive RT-QuIC replicates out of the total of eight replicates analyzed. The scale goes from 0 (all replicates were negative) to 1 (all replicates were positive). Fecal

homogenates were serially diluted (10^{-1} to 10^{-3}) and mouse rPrP was used as a substrate. **b.** The curves depict a representative RT-QuIC assay of fecal homogenates from Wisc-1-inoculated tg650 mouse #327 serially diluted (10^{-1} to 10^{-3}) using human rPrP substrate. The black arrow indicates the substrate replacement. The cut-off (threshold) was based on the average fluorescence values of negative control +5x SD used in this assay.

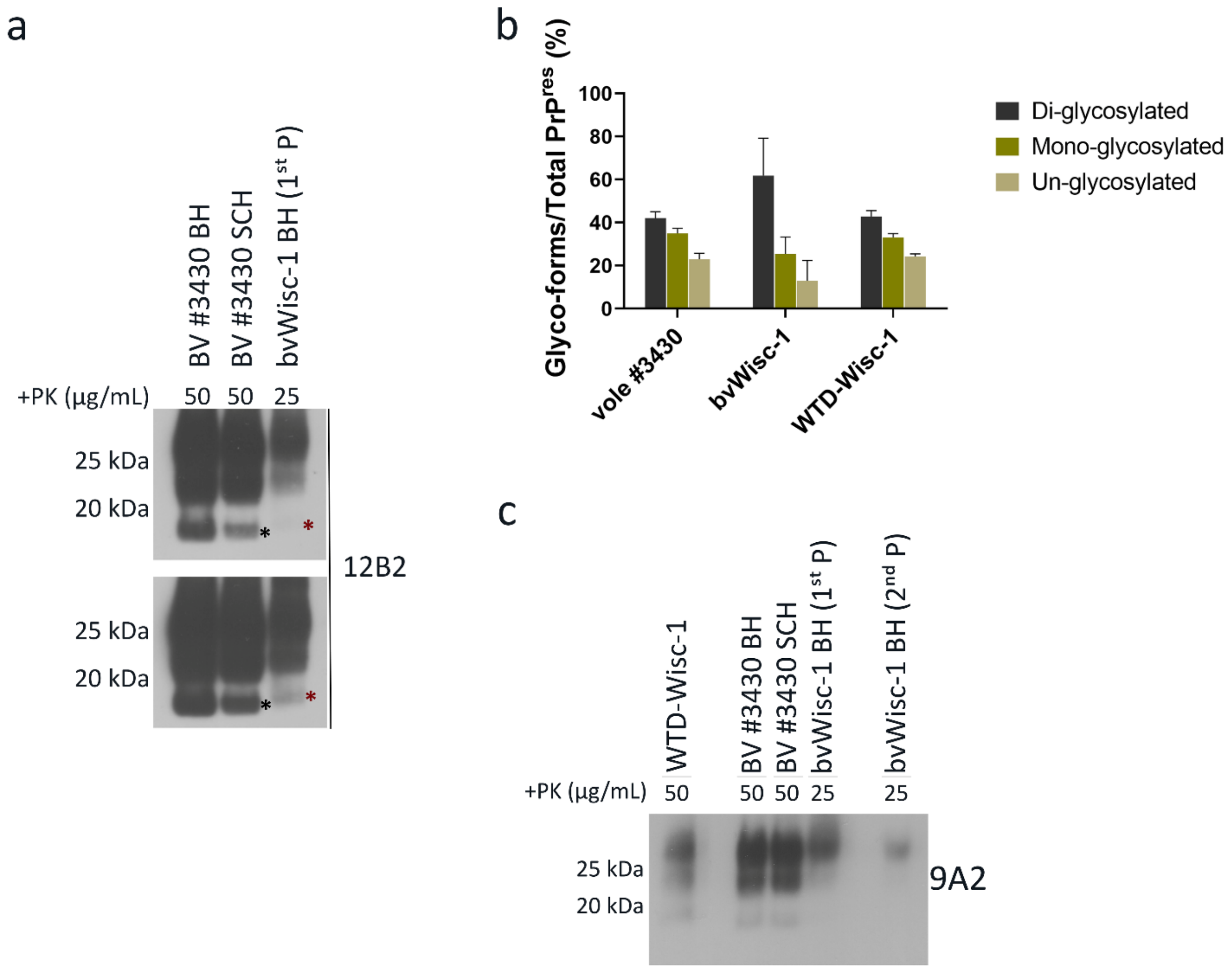

Supplementary Fig. 10

Transmission of CWD-tg650 fecal material to bank voles. 10% fecal homogenate from Wisc-1 inoculated tg650 mouse #327 was inoculated intracerebrally to bank voles. **a.** Western blot analyses of fecal homogenate-inoculated bank vole #3430 using brain homogenates (lane 1) and

spinal cord homogenates (lane 2) digested with 50 µg/mL of PK, as well as Wisc-1 passaged in bank voles (bvWisc-1) 1st passage (lane 3) digested with 25 µg/mL of PK. The western blot was probed with mAb 12B2. The amount of bvWisc-1 loaded on the gel was 20x that of bank vole #3430. The upper and lower panels are the same blots with different exposure times to better show the slower migration of the un-glycosylated band depicted in the bvWisc-1 1st passage. **b.** Quantification of the glycoform ratios of Wisc-1 isolate, bank vole #3430, and bvWisc-1 (1st and 2nd passage) prions.
